# p53, p63 and p73 expression and angiogenesis in keratocystic odontogenic tumors

**DOI:** 10.4317/jced.52843

**Published:** 2016-12-01

**Authors:** Soranun Chandrangsu, Kraisorn Sappayatosok

**Affiliations:** 1 Department of Oral Pathology, Faculty of Dentistry, Chulalongkorn University, Bangkok, Thailand; 2 Assistant Professor, Faculty of Dental Medicine, Rangsit University, Thailand

## Abstract

**Background:**

Keratocystic odontogenic tumors (KCOTSs) are odontogenic tumors previously referred to as odontogenic keratocysts. Several studies have reported that KCOT behavior is more like that of a benign neoplasm than a cyst. KCOTs are locally destructive and exhibit a high recurrence rate. The objective of this study is to characterize the expression of p53, p63 and p73 in KCOTs together with the relationship between their expression and KCOT angiogenesis and recurrence.

**Material and Methods:**

Standard indirect immunohistochemistry using monoclonal antibodies specific to human p53, p63, p73 and CD105 was performed in formalin-fixed paraffin-embedded tissue sections of 39 KCOT samples. Grading of p53, p63 and p73 immunohistochemical staining was divided into three groups, whereas microvessel density (MVD) was presented as the mean +/- standard deviation. Associations between p53, p63 and p73 expression and clinical-pathological parameters were analyzed by Fisher’s exact test, whereas associations among MVD levels, clinical and pathological parameters and p53, p63 and p73 expression were analyzed by the Mann-Whitney U test. Correlations among p53, p63, p73 and MVD levels were analyzed using Spearman’s correlation coefficients. For all analyses, *p*< 0.05 was considered to indicate statistical significance.

**Results:**

p53, p63 and p73 expression was noted in 23, 32 and 26 of 39 KCOT cases, respectively. The mean MVD was 26.7 ± 15.8 per high-power field. In addition, correlations between the expression levels of p53, p63, p73 and MVD in KCOT were examined. Statistically significant positive relationships were noted for all proteins (*p*<0.001).

**Conclusions:**

Three members of the p53 protein family are expressed in KCOTs, and their expression relates to angiogenesis in these tumors.

** Key words:**p53, p63, p73, angiogenesis, keratocystic odontogenic tumors.

## Introduction

Keratocystic odontogenic tumors (KCOTs) are odontogenic tumors that were previously termed odontogenic keratocysts. The origin of KCOTs is believed to be the remnant of the dental lamina (1). Several studies have reported that KCOT behavior is more like that of a benign neoplasm than a cyst (2). KCOTs are noted in approximately 11% of all cysts of the jaw (3). Sixty percent of the lesions are noted in people between 10 and 40 years of age. These tumors are most commonly noted in the mandible, especially in the body and ramus regions (4). KCOT patients may present with swelling, pain and discharge or may be asymptomatic. KCOT may have the potential for local destruction and atendency for multiplicity, especially when the lesion isassociated with nevoid basal cell carcinoma syndrome (NBCCS) (5,6).

KCOTs have a high recurrence rate, reportedly between 0% and 100% (7). Recurrence has been reported to be higher both in tumors associated with NBCCS (82%)(5) and in lesions arising from the mandible (8). Most recurrences will present within the first 5 to 7 years (9), although recurrences have been reported to occur 9 or more years after the initial treatment (10). Various factors support the high recurrence rate of KCOTs. The first factor is the KCOT growth pattern, which is locally destructive (5,11). The second factor is the KCOT genetic background (1). Growth of KCOT is controlled by the tumor suppressor gene, PTCH. PTCH will bind to SMO, forming a transmembrane receptor complex. This complex will inhibit the growth signal transduction of KCOTs. However, if sonic hedgehog (SHH) binds to PTCH, its ability to inhibit growth signal transduction is lost. If SMO is free from forming a complex with PTCH, it will activate the GLI1 protein, thus increasing the cell proliferation rate, which is a major contributing factor for the pathogenesis of KCOTs (1).

The third factor is evidence from histological studies of KCOTs, demonstrating that cells in the suprabasal layer of KCOTs have the potential to proliferate into the connective tissue layer. Moreover, mitotic figures are typically observed in this layer, supporting the proliferation capacity of the KCOT epithelial lining (5).

The development of tumors involves two types of genes during tumorigenesis: proto-oncogenes and tumor suppressor genes (12). Tumor suppressor genes play an essential role in inhibiting cell proliferation, which is normally inhibited or involves loss of function (12). The major tumor suppressor gene is Tp53,which is located on chromosome 17 (13) and acts during p53 protein synthesis (14). Numerous immunohistochemical studies including odontogenic cysts and tumors have identified the relationship between p53 and tumorigenesis, especially in KCOTs (14,15).

Many new genes have been discovered and classified in the p53 family, including Tp63. Tp63 generates p63, which exhibits 60% amino acid sequence similarity with the p53 protein (16). From a recent study, expression of ∆Np63 in benign odontogenic tumors is associated with high-risk recurrence more than in non-aggressive, benign odontogenic tumors with low-risk recurrence (17). Moreover, p63 is expressed in greater than 50% of malignant odontogenic tumors compared with benign odontogenic non-aggressive tumors (17). p73, another protein in the p53 family, is also mutated in human tumors (18).

When tumor growth exceeds some stage, the tumor forms new blood vessels to maintain its growth. The process begins with acceleration of protein synthesis, such as synthesis of vascular endothelial growth factor (VEGF). VEGF is released from the tumor and attach to the receptor on the vessel wall. Then, the vessel will release an intrinsic factor to stimulate more vessel formation (19).

In conclusion, tumor development requires many essential factors, genes and angiogenesis. According to several studies, a clear relationship is noted between p53, p63, and p73 expression and angiogenesis (15,20). However, none of the previous studies have reported the relationship between this tumor suppressor gene family and angiogenesis in KCOTs. Consequently, the purpose of this research was to study the expression of p53, p63, and p73 and the relationship between these proteins and angiogenesis in KCOTs.

## Material and Methods

In total, 39 KCOT specimens from biopsy or surgical specimens from the Department of Oral Pathology, Faculty of Dentistry, Chulalongkorn University, Srinakharinwiroj University and Rangsit University were obtained. Cases were excluded if the specimens included any other associated pathologies.

Histopathological slides were prepared using formalin-fixed, paraffin-embedded archival specimens. The tissue sections were cut 4μm thick, initially stained with hematoxylin and eosin (H&E) and examined under a light microscope by 2 oral pathologists to confirm the diagnosis.

-Immunohistochemical technique

Paraffin-embedded blocks from the tumor and positive controls were cut 4μm thick, placed on lysine-coated slides and then processed using a standard immunohistochemical technique.

Monoclonal mouse anti-human p53, p63 (Dako, Denmark) and p73 protein (ABCAM, USA) were used. For angiogenesis assessment, monoclonal mouse anti-human CD105 antibody or Endoglin (Dako, Denmark) was used. The tissue sections slides were treated with a boiling solution of freshly prepared Tris-EDTA buffer (pH 9.0) in a microwave for 10 minutes. After cooling down to room temperature, the tissue sections were blocked from non specific reactions with normal goat serum at a dilution of 1:100 for 10 minutes. The sections were incubated in a moist chamber at 4°C overnight with primary antibody. Each primary antibody was used at a dilution of 1:100. Then, slides were rinsed in Tris-buffered saline twice before being treated with the Dako EnVision+System, (Product code: K4000, Dako North America Inc.) ata dilution of 1:100 for 60 minutes at room temperature.

The immunohistochemical reaction was visualized by developing the slides in 3,3’ diaminobenzidine tetrahydrocholride (Vector Laboratories, USA) and counterstaining with Mayer’s hematoxylin. Then, the tissue sections were dehydrated, cleared and mounted. The experiments were performed in triplicate.

The sections were evaluated under a Nikon Eclipse 800 microscope (Nikon Corporation, Japan). For p53,p63 and p73 immunore-activity, epithelial cells in the basal-parabasal, intermediate and superficial layers that were positive for p53, p63 and p73 with brown staining in the nucleus were exclusively scored. A mean percentage of positive cells was determined from the percentage of positive nuclei derived from the analysis of 100 cells in 10 random areas at 40X magnification. Positivity for p53, p63 and p73 was evaluated independently by two of the authors (KS and SC) who were blind to the clinical-pathological data. A semi-quantitative assessment of p53, p63 and p73 expression was performed by assigning cases to one of the three following categories: (a) score 0, when the stained cells were from 0 to <5% of the total cell population; (b) score 1, when the stained cells were from >5 to <50% of the total cell population; or (c) score 2, when the stained cells accounted for >50% of the total cell population.

Microvessel density (MVD) quantifications were performed using a Nikon Eclipse 800 microscope according to the method suggested by Weidner *et al.* (21). The definition of a microvessel used in the present study wasthe definition described previously (22). Three areas with the highest amount of vascularization (hotspots) were selected under a magnification of 10X. Microvessels were counted in each of the three fields at 40X magnification, and the mean density was reported. All slides were simultaneously evaluated by two observers using a double-headed microscope, and both observers were required to agree on each of the individual microvessels before they could be included in the count.

-Statistical analysis

For statistical analysis, the clinical-pathological parameters were grouped as follows: age below or above 37 years (the mean age of patients); male or female; maxilla or mandible; unilocular or multilocular radiolucency and local recurrence. The data were analyzed using SPSS software for Windows, version 22.0 (SPSS Inc., Chicago, IL, USA). MVD levels were expressed as the mean ± standard deviation (SD). Associations between p53, p63 and p73 expression and clinical-pathological parameters were analyzed by Fisher’s exact test, whereas associations among MVD levels, clinical-pathological parameters and p53, p63 or p73 expression were analyzed using the Mann-Whitney U test. Correlations between p53, p63, and p73 expression and MVD were analyzed using Spearman’s correlation coefficients. For all analyses, *p*< 0.05 was considered to indicate statistical significance.

The study was ethically approved by the Ethical Committee, Faculty of Dentistry, Chulalongkorn University, Thailand.

## esults

In total, 22 male (56.4%) and 16 female (43.6%) patients with a mean age of 37.1 ± 21.8 years were included in the study (range 7-84 years). The majority of lesions were located in the mandible (76.9%) and were demonstrated to have unilocular radiolucency (74.4%). Seventeen patients reported local recurrence (43.6%). The differential expression of p53, p63, and p73 and MVD levels in KCOTs are reported in  Table 1.

**Table 1:**
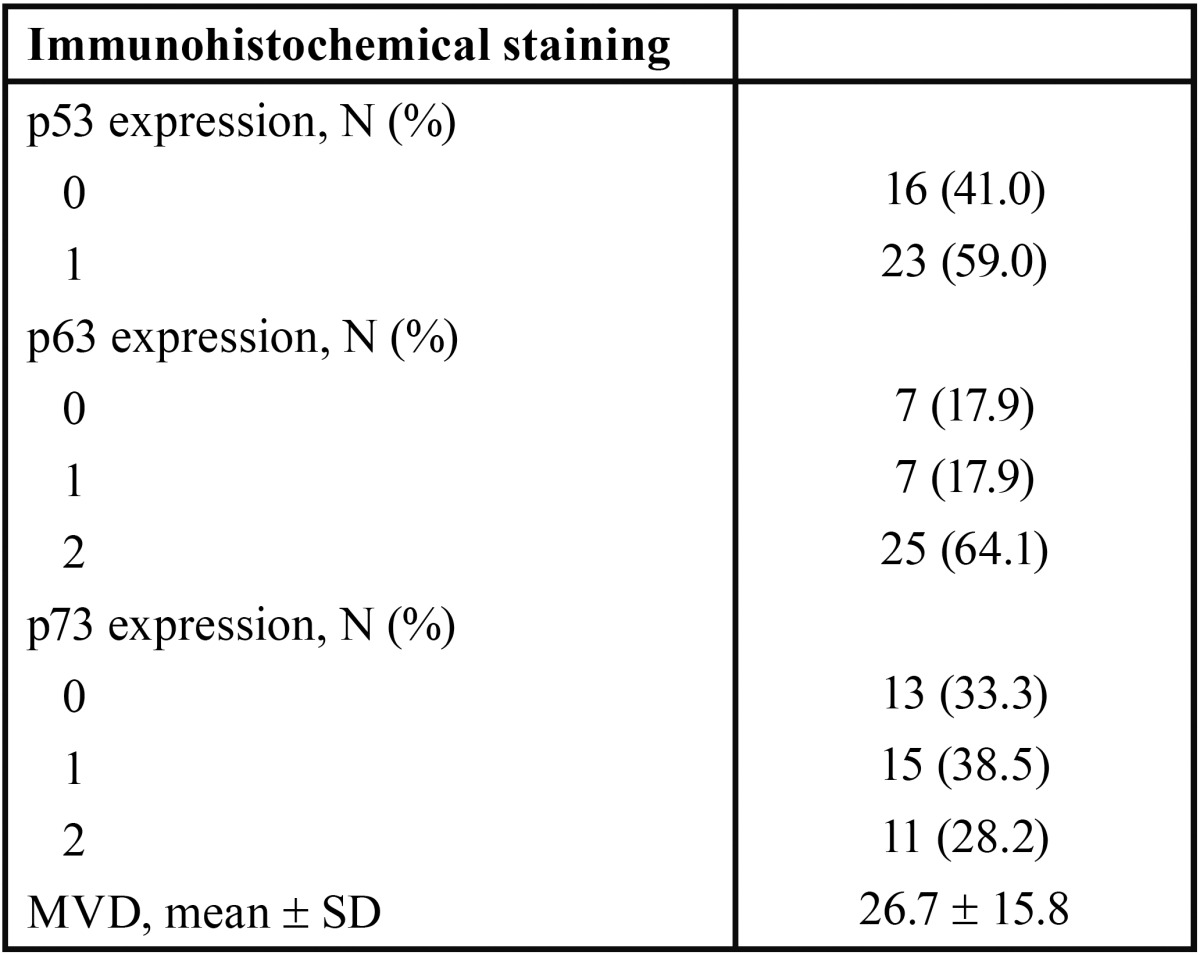
Differential p53, p63, and p73 expression and MVD levels in KCOTs.

**Figure 1:**
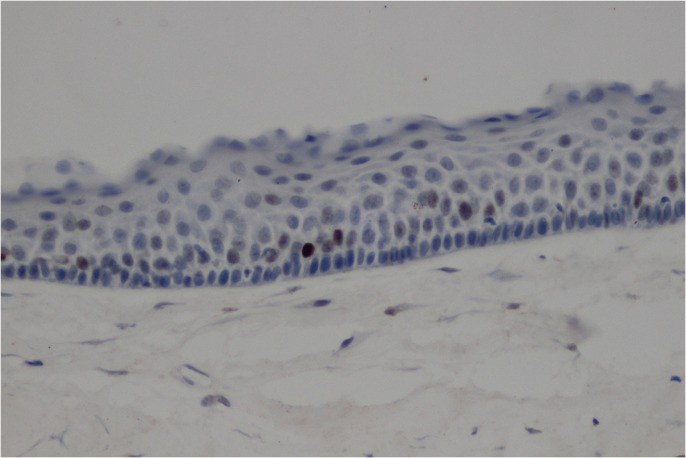
Representative photomicrographs (X200) of p53 staining in the basal cell layer of KCOT cystic epithelial lining.

p53 expression (Fig. 1) was observed in 59.0% of cases. The majority of these cases contained between 6 and 50% positive cells (score 1; 59.0%) followed by less than 5% of positive cells (score 0; 41.0%). No case exhibited greater than 50% of p53-positive cells.

**Figure 2:**
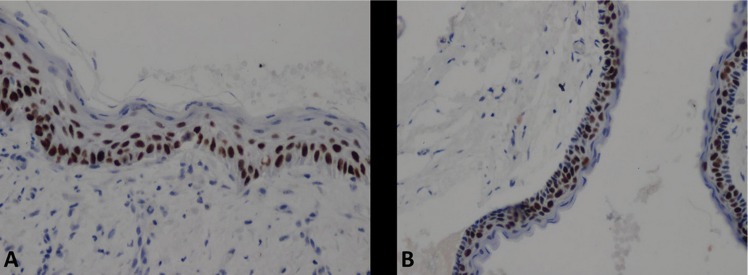
Representative photomicrographs (X200) of high p63 and p73 expression. A) p63 staining in basal and suprabasal cell layersof KCOT cystic epithelial lining (X200). B) p73 staining in all layers of KCOT cystic epithelial lining (X200).

p63 expression (Fig. 2A) occurred more frequently (82.0%) than p53. Greater than 60% of cases exhibited greater than 50% p63-positive cells (score 2; 64.1%), whereas tumors exhibiting between 6 and 50% positive cells (score 1; 17.9%) were observed with equal frequency as those withless than 5% positive cells (score 0; 17.9%). p73 expression (Fig. 2B) appeared to be less frequent (66.7%) than that of p63 but more frequent than that of p53. The majority of cases contained between 6 and 50% positive cells (score 1; 38.5.0%), followed by less than 5% positive cells (score 0; 33.3%) and greater than 50% positive cells (score 2; 28.2%).

**Figure 3:**
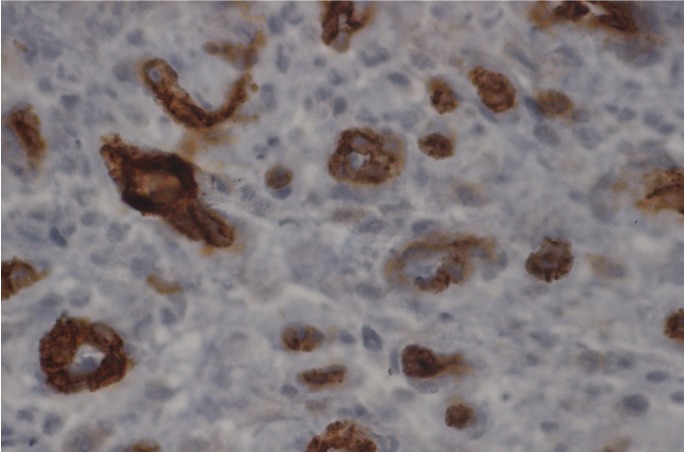
Representative photomicrographs (X200) of p53 staining in the basal cell layer of KCOT cystic epithelial lining.

The microvessel density representing angiogenesis is presented in figure 3. The mean MVD value was 26.7 ± 15.8 per high-power field (HPF). In addition, the correlations in expression levels among p53, p63, and p73 as well as MVD levels in KCOTs were examined. Statistically significant positive relationships were noted for all proteins (*p*< 0.001,  Table 2).

**Table 2:**
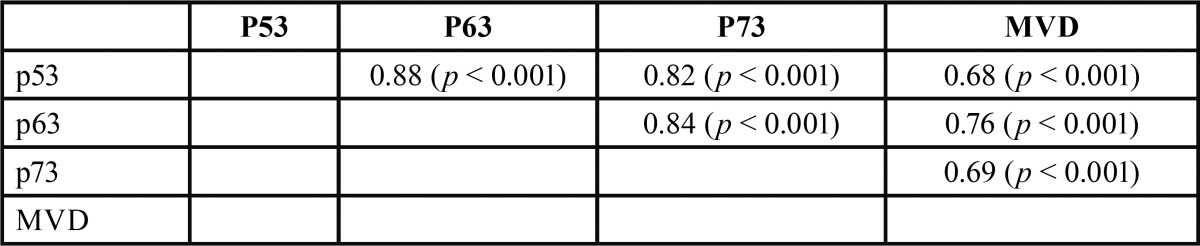
Correlations between p53, p63, and p73 expression and MVD levels in KCOTs, Spearman’s rho (*p*-value).

-Associations between p53, p63 and p73 expression and clinical-pathological parameters of KCOT patients

For the association analysis of p53, p63 and p73 expression and clinical-pathological parameters, cases were divided into 2 groups: the low expression group (cases with score 0) and the high expression group (cases with score 1 and 2). The results are presented in  Table 3. Increased p53, p63 and p73 expression was significantly associated with local recurrence (*p* = 0.001, 0.012 and 0.017, respectively). On the contrary, no statistically significant relationship was noted between p53, p63 and p73 expression and other clinical-pathological parameters, including age, gender, location and radiographic features.

**Table 3:**
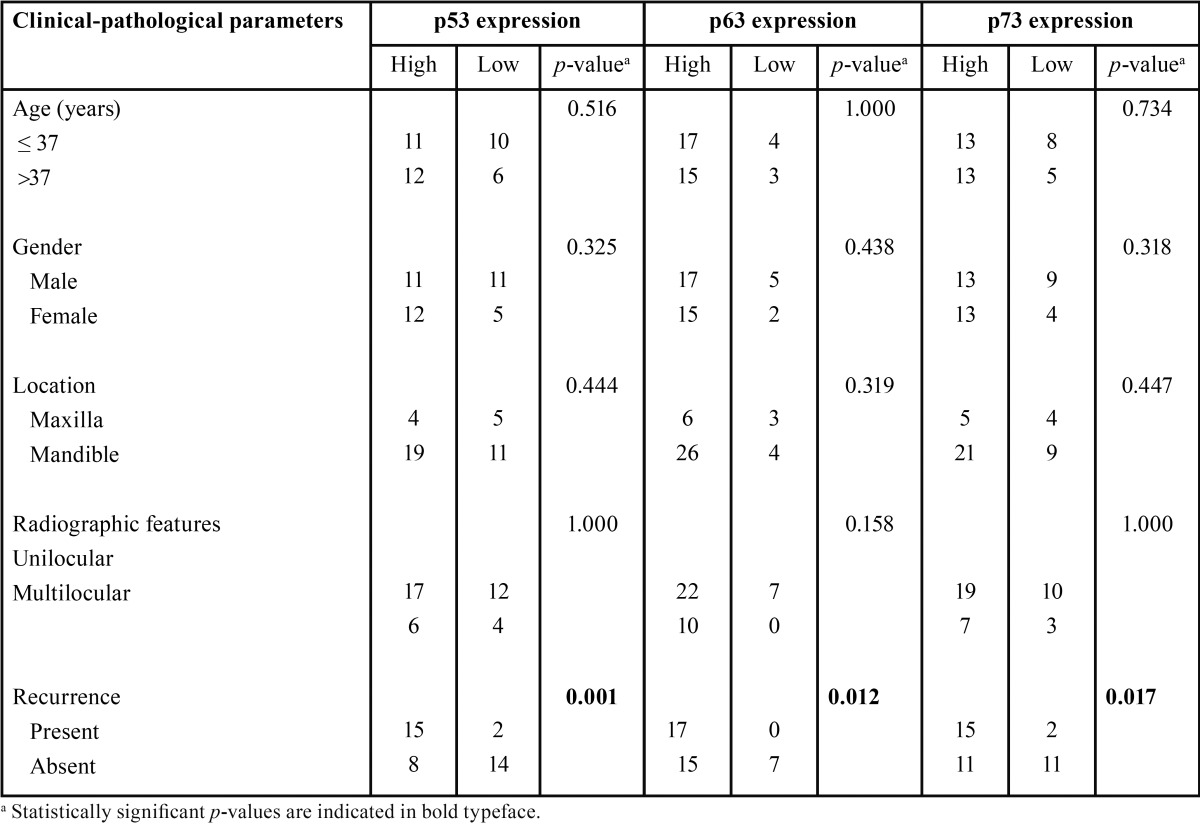
Relationship between p53, p63 and p73 expression and clinical-pathological parameters of KCOT patients.

-Associations between MVD expression level, clinical-pathological parameters and p53, p63 and p73 expression in KCOT patients

Statistically significant differences in MVD expression level were demonstrated with regard to local recurrence and p53, p63 or p73 expression (*p*< 0.05). Higher MVD levels were detected in the cases with local recurrence (*p* = 0.012), high p53 expression (*p*< 0.001), high p63 expression (*p* = 0.006) and high p73 expression (*p*< 0.001). No statistically significant relationship was noted between MVD level and other clinical-pathological parameters, including age, gender, location and radiographic features, ( Table 4).

**Table 4:**
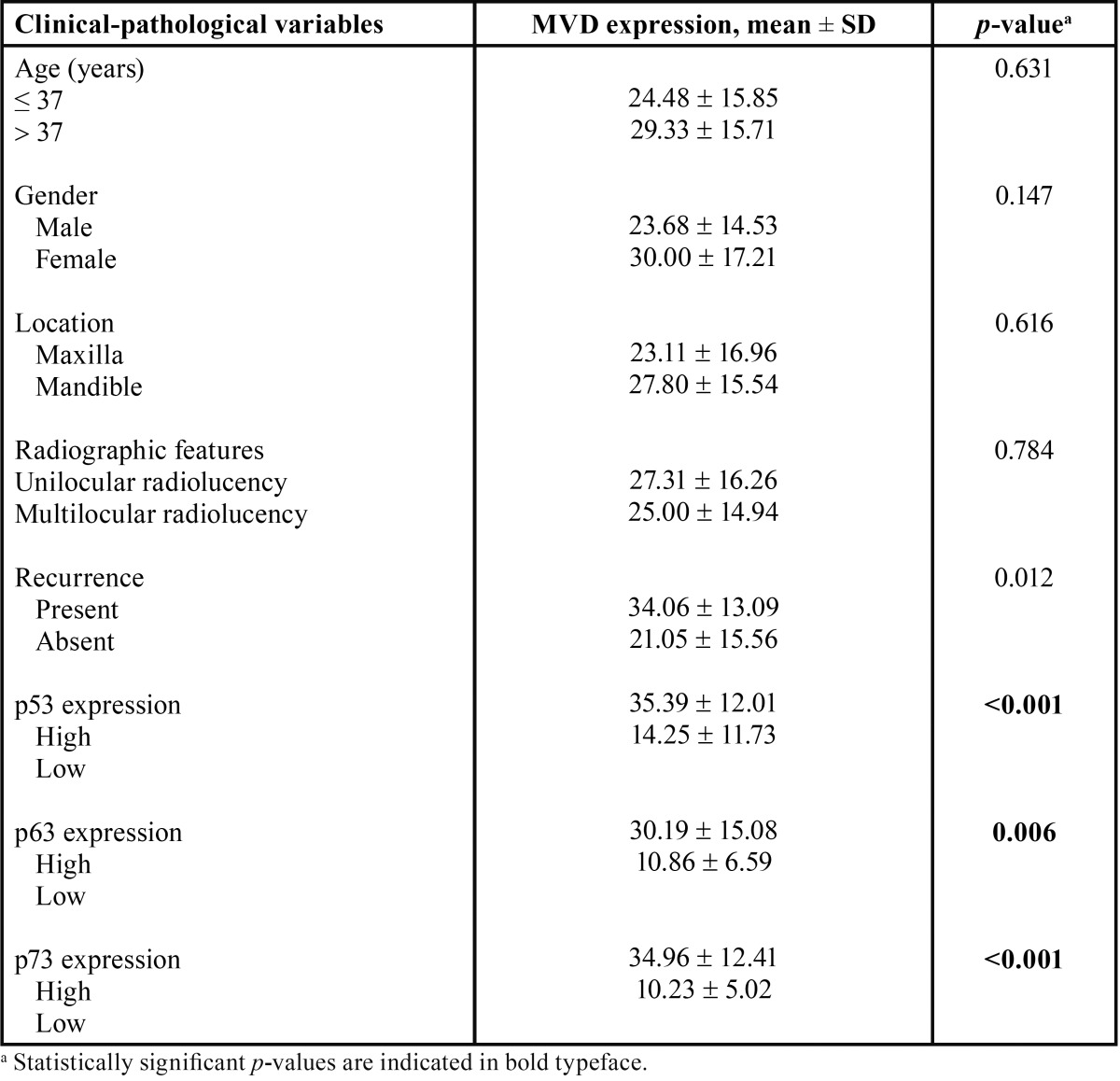
Summary of study variables grouped by MVD expression in KCOTs.

## Discussion

In previous studies, KCOTs were considered to be tumors rather than cysts due to their invasiveness and ability to form new vessels (21). These lesions appear to have an intrinsic growth potential and a marked tendency to recur. The epithelium of KCOTs is relatively thin and fragile, which enables budding of the basal layer into the underlying connective tissue and gives rise to what have been termed daughter cysts (1).

p53 mutations are amongst the most common genetic alterations in human cancer. Several studies demonstrate that p53 expression correlates with tumor grade and recurrence (23). Alterations in the p63 gene have been proposed as one of the factors responsible for tumorigenesis in oral cancer. Thus, p63 gene mutations may be considered a prevalent finding in malignancies of the oral mucosa (24). p73, which appears to be less studied, also exhibits a relationship with tumor invasiveness and response to treatment in oral squamous cell carcinoma. Many studies have demonstrated that mutations in the p53, p63 and p73 tumor suppressor genes are frequently detected in human cancers (25). Our study demonstrated p53, p63 and p73 expression in the epithelial layer of KCOTs in 23, 32, and 26 of 39 cases, respectively. This expression correlates with tumor recurrence, which has not been demonstrated in previous studies. Positive p53, p63 and p73 staining was primarily noted in the basal and parabasal layers, whereas the superficial layer was negative. These results are consistent with the study performed by Muzio (20) and Varsha (26), whereas the study from Gurgel (15) observed p53 expression mainly in the suprabasal layer and p63 expression in all of cystic epithelial lining, including the superficial layer. Our study also contrasts with the study by Li, noting that p53 expression primarily occurred in the suprabasal layer (27). Interestingly, our study is the first to demonstrate p73 expression and recurrence in KCOTs; meanwhile, p73 expression is associated with recurrence of colon carcinoma (28).

One of the most important factors in tumor growth is angiogenesis. Angiogenesis is typically defined as the growth of new blood vessels. Angiogenesis in oral squamous cell carcinoma is related to tumor recurrence (29). Our study shows that KCOT recurrence relates with angiogenesis and p53 protein family expression, which supports the idea of the tumor nature of KCOTs. Moreover, our results correspond with those from the study of Gadbail *et al.* (30), indicating numerous microvessels expressing CD105 with intense immunoreactivity in KCOTs.

The results of this study suggest that p53, p63 and p73 expression might reflect the replication potential of epithelium that may favor tumorigenesis. This finding supports the hypothesis that KCOTs exhibit more neoplastic characteristics (17,20). Moreover, the relationship between p53, p63 and p73 expression and angiogenesis could suggest the proliferative ability and modulation of tumor angiogenesis, which may support the tumor nature of KCOTs. In the next World Health Organization (WHO) classification of odontogenic cysts and odontogenic tumors, regardless of whether KCOTs are reclassified as cysts or tumors, they should be regarded as aggressive lesions given that our study confirms their neoplastic nature through p53 protein family expression and angiogenesis.

## Conclusions

In KCOTs, p53, p63 and p73 expression and CD105-positive microvessels were observed. Moreover, p53, p63 and p73 expression correlated with MVD in KCOTs. This evidence supports the theory of the highly intrinsic growth potential of the KCOT epithelium and tumor angiogenesis. Thus, it can be concluded that p53, p63 and p73 expression and increased angiogenesis may contribute to the locally aggressive and invasive behaviors of KCOTs.
